# Successful surgical management of esophageal leiomyoma presenting with gastroesophageal reflux disease symptoms: A case report

**DOI:** 10.1016/j.ijscr.2024.110746

**Published:** 2024-12-15

**Authors:** Abdalrahman N. Herbawi, Saif K. Azzam, Ibrahim AboGhayyada, Osama Hroub, Kareem Ibraheem, Badawi Eltamimi

**Affiliations:** a Faculty of Medicine, Palestine Polytechnic University, Hebron 90200, Palestine; b Gastroenterology Department, Al Ahli Hospital, Hebron 90200, Palestine; c Palestinian Clinical Research Center, Bethlehem, Palestine.

**Keywords:** Esophageal leiomyoma, Esophageal mass, Video-assisted thoracoscopic surgery (VATS), Gastroesophageal refelx disease, Endoscopic ultrasound (EUS)

## Abstract

**Introduction:**

Esophageal leiomyoma is the most common benign submucosal mesenchymal tumor of the esophagus, typically asymptomatic but can cause symptoms such as dysphagia, chest pain, or regurgitation when large. Diagnosis is often incidental, confirmed by imaging techniques like computed tomography (CT) and endoscopic ultrasound (EUS), with surgical enucleation being the standard treatment.

**Presentation of case:**

A 28-year-old male presented with a one-year history of persistent epigastric discomfort and gastroesophageal reflux disease (GERD) symptoms unresponsive to proton pump inhibitors. Chest radiograph and CT scan revealed a well-defined submucosal mass in the esophagus. Upper gastrointestinal endoscopy and EUS confirmed the lesion's benign nature. Fine-needle aspiration biopsy showed spindle-shaped cells, confirming esophageal leiomyoma. The patient underwent minimally invasive tumor resection via video-assisted thoracoscopic surgery (VATS), with a smooth postoperative recovery.

**Discussion:**

Esophageal leiomyomas are rare, often asymptomatic, and may present with nonspecific symptoms if large. CT and EUS are key diagnostic tools, and minimally invasive surgery, such as VATS, is the preferred treatment for larger tumors due to shorter recovery times and fewer complications. Early identification and appropriate surgical intervention are crucial for optimal outcomes.

**Conclusion:**

Esophageal leiomyoma should be considered in patients with GERD-like symptoms unresponsive to therapy. Early imaging, endoscopic evaluation, and minimally invasive surgery provide excellent outcomes, with regular follow-up recommended to monitor for recurrence.

## Highlights

• Esophageal leiomyoma can mimic persistent GERD symptoms, requiring advanced diagnostics.
• Endoscopic ultrasound (EUS) is vital for diagnosing submucosal esophageal tumors.
• Video-assisted thoracoscopic surgery (VATS) ensures safe and effective tumor enucleation.
• Minimally invasive techniques improve outcomes and reduce recovery time for esophageal leiomyomas.
• Early surgical intervention resolves symptoms and prevents potential complications.

## 1 Introduction

Esophageal leiomyoma, although rare, is the most common benign submucosal mesenchymal tumor (SMT) of the esophagus, arising from smooth muscle cells. Typically, esophageal leiomyomas present as solitary tumors [1]. Most are asymptomatic and measure <5 cm, often discovered incidentally during routine examinations. Larger tumors may cause symptoms like dysphagia, chest pain, heartburn, unexplained retrosternal discomfort, and regurgitation [2]. Diagnosis is often incidental during upper gastrointestinal (GI) assessments, confirmed through computed tomography (CT) and endoscopic ultrasound (EUS). Surgical enucleation, especially using minimally invasive techniques, is increasingly preferred due to its safety and effectiveness. Most leiomyomas originate in the inner circular muscle layer and are located in the distal and mid-thoracic esophagus, particularly near the esophagogastric (EG) junction [3]. This paper discusses esophageal leiomyoma, its diagnosis, management, and the benefits of minimally invasive techniques like VATS for treatment. This article has been reported in line with the SCARE 2023 guideline, which updates the consensus Surgical Case Reports (SCARE) guidelines [4].

## 2 Case presentation

**Fig. 1:**
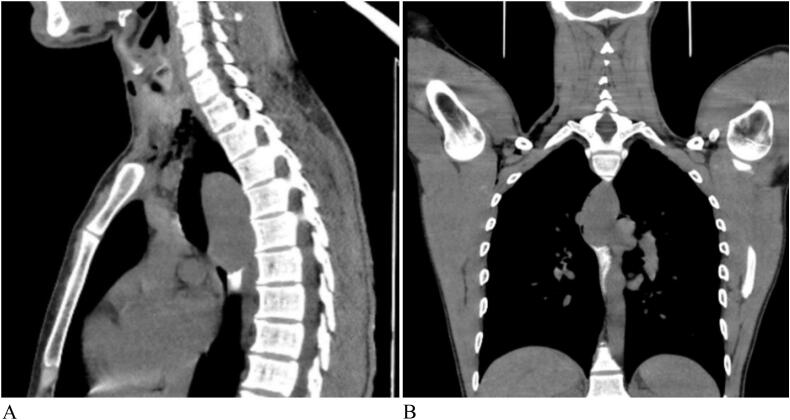
CT image showing a 3.4 cm × 2.5 cm × 5.3 cm soft tissue mass confined to the esophageal wall, with no evidence of invasion into surrounding structures, suggesting a submucosal origin.

A 28-year-old male presented with a one-year history of persistent epigastric discomfort and GERD symptoms that were minimally responsive to proton pump inhibitors (PPIs). The patient denied weight loss, dysphagia, or systemic symptoms. His medical history was unremarkable, with no prior surgeries or systemic diseases. Physical examination findings were normal. A chest radiograph revealed mediastinal widening, raising suspicion for a mediastinal mass. A contrast-enhanced CT scan of the chest showed a 3.4 × 2.5 × 5.3 cm, well-defined, poorly enhancing submucosal mass within the esophagus, confined to the esophageal wall without invasion of surrounding structures (Fig. 1A: Sagittal view; Fig. 1B: Frontal view).

**Fig. 2:**
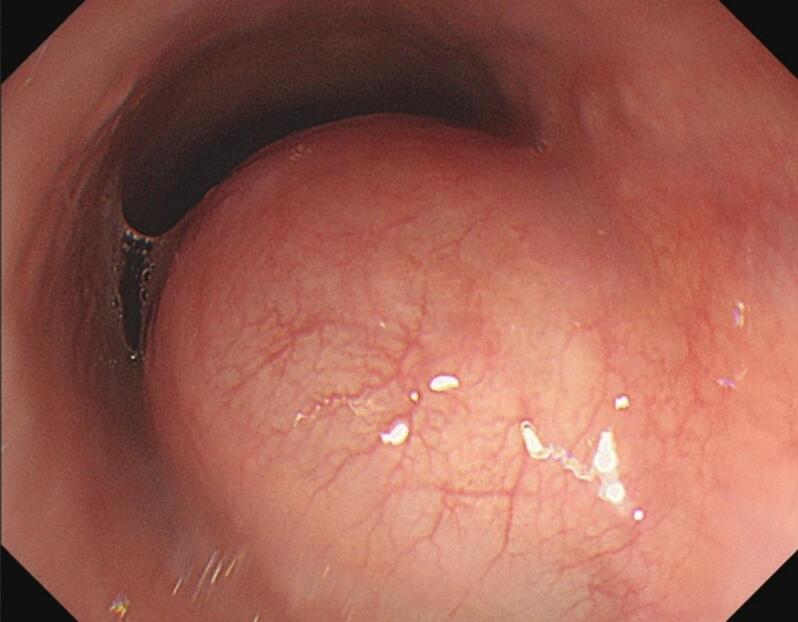
An endoscopic image revealed a protruding submucosal lesion with a smooth surface.

**Fig. 3:**
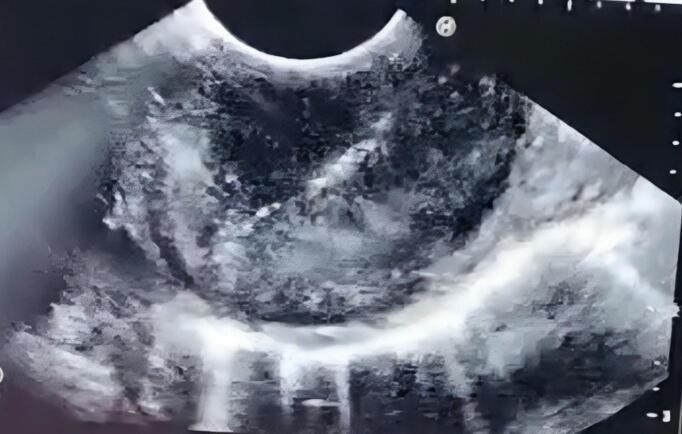
EUS image showing a hypoechoic mass originated from the muscularis propria layer.

Upper GI endoscopy identified a smooth, protruding submucosal lesion located 6 cm from the gastroesophageal junction, without ulceration or mucosal breach (Fig. 2). EUS revealed a hypoechoic mass originating from the muscularis propria layer, consistent with a benign mesenchymal tumor (Fig. 3). Fine-needle aspiration (FNA) biopsy showed spindle-shaped cells arranged in interlacing fascicles, confirming the diagnosis of esophageal leiomyoma. The patient underwent minimally invasive tumor resection using VATS. Under general anesthesia and single-lung ventilation, small thoracoscopic incisions provided access to the thoracic cavity. The tumor was completely enucleated, and intraoperative findings confirmed its benign nature. Postoperatively, the patient recovered smoothly, with a chest tube placed to ensure proper lung re-expansion. He was discharged on postoperative day two. At the six-week follow-up, the patient reported complete resolution of GERD symptoms, and a repeat CT scan showed no evidence of recurrence.

## 3 Discussion

Benign esophageal tumors are rare, accounting for <1 % of all esophageal tumors. The most common type of benign esophageal tumor is leiomyoma, while other benign esophageal tumors are typically cysts or polyps [5]. Esophageal leiomyomas are generally slow-growing and have a low risk of malignancy. In some instances, they can become quite large, exceeding 10 cm in diameter, and are then classified as giant leiomyomas, often presenting as a mediastinal mass. These tumors are most frequently found in the lower two-thirds of the esophagus, where smooth muscle cells are more concentrated. Large leiomyomas in the distal esophagus may occasionally compress the gastric cardia [5].

When symptomatic, patients usually report non-specific symptoms that persist over time. Common symptoms include dysphagia, pain, and weight loss [6]. In our case, the patient experienced epigastric discomfort and gastroesophageal reflux disease (GERD) for one year.

To assess esophageal leiomyomas, imaging techniques like barium esophagography, endoscopy with endoscopic ultrasound (EGD/EUS), chest X-ray, CT, and MRI are employed [7]. Plain radiographs have limited sensitivity and specificity, often requiring a larger mass to be visible [8]. CT scans of the chest typically show a mass originating from the esophagus, usually without mediastinal lymphadenopathy. For giant leiomyomas, the tumor may extend outward from the esophageal lumen, creating a soft tissue shadow in the mediastinum, which can sometimes lead to a misdiagnosis of a mediastinal mass [9].

Conventional endoscopy is helpful in identifying lesions and differentiating them from polyps, cancer, and other conditions, though it cannot provide detailed information on lesion size, origin, or relationship to nearby organs. Additionally, it may not distinguish between esophageal leiomyomas and lesions caused by external compression or other submucosal abnormalities [10].

Endoscopic ultrasound (EUS) is the primary diagnostic tool for leiomyoma, as it combines endoscopic and ultrasound technology to evaluate the surface shape of gastrointestinal lesions, their layer of origin, depth of invasion, and structural features [11].

The size and location of a leiomyoma are key factors in determining the most suitable surgical approach. Endoscopic techniques are recommended for small, pedunculated tumors (2–4 cm) that originate from the muscularis mucosae [12]. According to a study by Wile RK et al. [16], treatment options for symptomatic leiomyomas under 5 cm include enucleation through open surgery [13,14] or video-assisted thoracoscopy (VATS) [15]. VATS is especially advantageous for larger leiomyomas (over 5 cm), as it allows concurrent endoscopic visualization, which is beneficial in difficult-to-access regions like the gastroesophageal junction (GEJ) [16].

In cases of leiomyomas in the lower esophagus near the GEJ, a left-sided VATS approach is preferred over laparoscopic methods, offering improved exposure and access to the distal esophagus. Additional exposure can be achieved by dissecting to the hiatus and using crural traction sutures [17].

During extra-mucosal excision or enucleation, a longitudinal incision is made in the outer esophageal muscle, and the leiomyoma is carefully separated from the mucosa. Thoracoscopic approaches offer benefits, including shorter hospital stays, fewer pulmonary complications, and reduced wound pain compared to open surgery. Endoscopic submucosal dissection and enucleation are also becoming more common, with some centers using Da Vinci robot-assisted thoracoscopy. Typically, the muscle layer is closed following myotomy and enucleation, although large myotomies or extra-mucosal defects may sometimes be left open without complications. Esophageal resection is generally reserved for very large tumors, while asymptomatic tumors under 1 cm are managed with regular follow-up [5].

Other minimally invasive techniques involve using endoscopy combined with balloon dilation assistance, in which an intraluminal balloon is inflated to push the tumor away from the esophageal wall, facilitating thoracoscopic resection [18].

After tumor enucleation, it is essential to check for mucosal breaches via direct inspection or by inflating air underwater. If a breach is found, it should be repaired, and the patient should abstain from oral intake for five days [17].

## 4 Conclusion

This case emphasizes the importance of considering esophageal leiomyoma in patients with persistent gastroesophageal reflux disease symptoms unresponsive to medical therapy. Early imaging, endoscopic evaluation, and multidisciplinary management are essential for timely diagnosis and treatment. Minimally invasive surgical techniques, such as VATS, provide excellent outcomes and symptom resolution in patients with benign esophageal tumors.

## Patient consent

Written informed consent was obtained from the patient for the publication of this case report.

## Informed consent

Written informed consent was obtained from the patient for the publication of this case report and accompanying images.

## Ethical approval

Ethical approval was not applicable for this study, as our institution's IRB committee at Palestine Polytechnic University does not mandate approval for reporting individual cases or case series.

## Funding

This research did not receive any specific grants from funding agencies in the public, commercial, or not-for-profit sectors.

## Author contribution

Abdalrahman N. Herbawi, Osama Hroub: Conceptualization, case analysis, manuscript writing, and editing.

Abdalrahman N. Herbawi, Ibrahim AboGhayyada, Kareem Ibraheem, Osama Hroub: Data collection, literature review, and manuscript drafting.

Badawi Eltamimi, Saif Azzam, Abdalrahman N. Herbawi, Kareem Ibraheem: Clinical management of the patient, data interpretation, and manuscript revision. All authors have read and approved the final manuscript and agree to be accountable for all aspects of the work.

## Guarantor

Kareem Ibraheem is the guarantor for this study, taking full responsibility for the research and its outcomes. Kareem Ibraheem had access to all the data and made the final decision to publish the study.

## Research registration number

None.

## Declaration of competing interest

The authors have no conflict of interest to declare.

## Data Availability

The data used to support the findings of this study are included in the article.
